# Vessel Density Changes on Optical Coherence Tomography Angiography after Vascular Endothelial Growth Factor Inhibitor Treatment for Diabetic Macular Edema

**DOI:** 10.4274/tjo.galenos.2020.81592

**Published:** 2020-12-29

**Authors:** Kai Xiong Cheong, Shu Yen Lee, Marcus Ang, Kelvin Yi Chong Teo

**Affiliations:** 1 Singapore Eye Research Institute, Singapore National Eye Centre, Singapore

**Keywords:** Optical coherence tomography angiography, vessel density, superficial capillary plexus, deep capillary plexus, vascular endothelial growth factor inhibitor, treatment response

## Abstract

**Objectives:**

To evaluate the changes in macular vessel density after treatment with vascular endothelial growth factor (VEGF) inhibitors in center-involving diabetic macular edema (DME) and to compare these changes between anatomical responders and non-responders.

**Materials and Methods:**

This retrospective study included 22 eyes with center-involving DME. All eyes had 3 consecutive administrations of VEGF inhibitors. Optical coherence tomography (OCT) and OCT angiography (OCTA) of the macula with manual adjustment of segmentation lines were performed at baseline and after treatment. Vessel density in the central and parafoveal regions of the superficial and deep capillary plexus (SCP/DCP) were measured at baseline and after treatment. Vessel density and changes therein were compared between anatomical responders and non-responders as defined by changes in central subfield thickness (CST).

**Results:**

Overall, there were no significant differences in vessel density in the central and parafoveal regions of the SCP and DCP after treatment compared to baseline. After categorization by anatomical response, 12 eyes were responders (CST decreased by 173.7±47.7 μm) and 10 eyes were non-responders (CST increased by 20.8±38.9 μm) (p<0.0001). There were no corresponding significant differences between responders and non-responders in SCP and DCP vessel density or changes therein after treatment.

**Conclusion:**

There were no significant changes in macular vessel density after the early stages of VEGF inhibitor treatment for DME, and there was no relationship with the anatomical response. The effect of VEGF inhibitors in DME treatment may not be related to increasing vessel density.

## Introduction

Diabetic macular edema (DME) is a major cause of visual impairment in patients with diabetes mellitus and it occurs as a result of the breakdown of the blood retinal barrier due to metabolic changes associated with hyperglycemia.^1^ The current treatment for DME targets vascular endothelial growth factor (VEGF), which has been identified as the most important factor in the pathogenesis of DME.^2^ However, while there is often a functional improvement after the resolution of DME with VEGF inhibition, ischemic changes may still result in irreversible vision loss in the absence of edema.

Assessment of the perfusion status of the macula, which is an important prognostic factor in DME, requires fundus fluorescein angiography (FA).^3,4^ FA requires the administration of fluorescein dye. FA is invasive and relatively more time consuming compared with optical coherence tomography angiography (OCTA).^3,4^ OCTA is a relatively new, non-invasive and rapid method of producing high-resolution and depth-resolved images of the retinal vasculature without the intravenous administration of dye.^5,6,7,8,9,10^ Layer-by-layer imaging can be performed on OCTA to assess the superficial and deep capillary plexuses (SCP/DCP) separately. En face images showing vascular changes on OCTA can be correlated with corresponding structural changes on OCT B-scans.^5,6,7,8,9,10^ OCTA is also easier to perform on sequential visits compared with conventional FA.^5,6,7,8,9,10^

In the assessment of diabetic retinopathy (DR) and DME, OCTA attempts to provide various quantitative parameters including vessel density and foveal avascular zone (FAZ) area.^11,12,13,14,15,16,17,18,19,20,21,22,23,24,25,26,27^ Of interest in this study is vessel density on OCTA, which is a quantification of the number of vessels in a region of interest.^23^ Many studies have reported decreased vessel density in the SCP and DCP in eyes with DR and DME compared with normal controls.^11,12,13,14,16,17,19,21,22^ This decrease is also more consistent in the DCP than the SCP.^13,16,20^ Notably, the changes in these OCTA parameters have been reported in diabetic patients without clinical signs of DR, which suggests a potential role of OCTA parameters in demonstrating early microvascular alterations in the capillary plexuses.^28,29^ In a recent prospective study, vessel density of the SCP and DCP were reported to predict the progression of DME and DR, respectively.^21^

However, the effect of VEGF inhibitor on macular vessel density in DME treatment remains controversial. While some studies reported an increase in vessel density after VEGF inhibitor treatment,^20,22^ others reported no change in vessel density in both the DCP and SCP despite reductions in edema and retinal thickness after treatment.^17,18,19,25^ It was also reported that certain eyes may not respond to VEGF inhibitors and demonstrate lower vessel density in the DCP but not the SCP.^16,20^ Damage to the DCP could thus be a useful predictor of response to VEGF inhibitor treatment in DME.^16,20^ Identification of factors associated with response and non-response to VEGF inhibitors is important because non-responders often require more treatments, which in turn increases cost and poses a significant burden on patients.^30^ In addition, delayed resolution of macular edema may cause photoreceptor damage that is irreversible.^31^

The equivocal findings in prior studies have resulted in the lack of widespread clinical use of OCTA in assessing DME. These inconsistencies can be attributed to the inherent shortcomings of OCTA. These include inaccurate segmentation and difficulty in obtaining vascular quantification as a result of distorted anatomy in diseased states.^32^ Furthermore, there is no consensus regarding the interpretation of DME features such as cysts and non-perfusion areas on OCTA.^26^

This study aimed to evaluate changes in macular vessel density in the central and parafoveal regions at the level of the SCP and DCP after 3 consecutive intravitreal VEGF inhibitor treatments in patients with treatment-naïve DME by comparing pre- and posttreatment OCTA images. Meticulous manual adjustment of the segmentation lines in each OCTA scan was performed when necessary to ensure accuracy and to allow quantification of the macular vessel density with the in-built software. Macular vessel density in the SCP and DCP and changes therein were subsequently compared between anatomical responders and non-responders.

## Materials and Methods

We performed a retrospective comparative study. All subjects had treatment-naïve center-involving DME diagnosed by a trained retina specialist with fundus slit-lamp biomicroscopy and OCT. All eyes had 3 consecutive administrations of VEGF inhibitors at least 30 days apart. A trained retinal specialist reviewed all the participants.

The inclusion criteria were treatment-naïve center-involving DME eyes with a central subfield thickness (CST) of 250 µm or greater,^33^ no previous documented DME, and adequate media clarity to obtain OCT and OCTA images. Exclusion criteria were significant ocular media opacity affecting the quality of the ophthalmic imaging, clinical evidence of retinal disease apart from DR, previous retinal surgery, and previous DME treatment.

Response was defined anatomically as a 10% decrease in CST from baseline.^33^ The DRCR Network has established that a change in OCT thickness of 10% or more is indicative of a real change in thickness that can be considered in the decision to continue or initiate treatment.^34^ Spectral-domain OCT and OCTA were performed at baseline and after the 3 VEGF inhibitor treatments. The study was conducted at the Singapore National Eye Centre, Singapore Health Services, Singapore. The study was approved by the Institutional Review Board and conformed to the tenets of the Declaration of Helsinki.

### Optical Coherence Tomography Angiography

The Triton (Topcon DRI OCT Triton Swept Source OCT; Topcon, Tokyo, Japan) features a wavelength of 1050 nm, an A-scan rate of 100000 A-scans per second, and an axial and transversal resolution of 8 and 20 µm in tissue, respectively. The scanning area was captured in 3x3 mm sections centered on the fovea. An active eye tracker was employed to reduce motion and blinking artifacts during OCTA.

The OCTA images were obtained with a minimum signal strength index of 50 and above and a quality score of 40 and above. The OCTA images were also assessed to look for blurriness, localized weak signals or signal loss, irregular vessel patterns and disc boundaries due to motion artifacts, and off-centered scans. The OCTA images were processed by the OCT Angiography Ratio Analysis (OCTARA) detection software.

### OCTA Segmentation

Automatic segmentation lines were used to divide the retinal capillary plexus into the SCP and DCP layers. The SCP was defined as the region between the vitreoretinal interface and the outer border of the ganglion cell layer. It was segmented with one boundary at 2.6 µm below the internal limiting membrane  and the other 15.6 µm below the inner plexiform layer (IPL). The DCP, defined as the region between the inner border of the IPL and the outer border of the outer plexiform layer, was automatically segmented with the boundaries set at 15.6 µm and 70.2 µm beneath the IPL, respectively.

The accuracy of the automatic segmentation lines was verified visually and independently by experienced graders (K.Y.C.T. and K.X.C.) by examining each B-scan image. Visual verification was necessary because large intraretinal cysts in DME often spanned multiple layers and this frequently caused segmentation errors, especially in the IPL, which is the layer that differentiates the SCP and DCP. Inaccurate segmentation was defined if the border between the SCP and DCP was not located within the range of the IPL. Segmentation errors were manually corrected by both graders using the built-in OCTARA software and vessel density was recalculated based on the new segmentation boundaries. Segmentation was deemed satisfactory when both graders agreed that the lines correlated to the correct anatomical layer.

The segmentation boundaries for all eyes in the SCP and DCP were assessed and manually corrected on two separate occasions by the same experienced grader (K.Y.C.T.). The resultant measurements were compared to calculate the intraclass correlation coefficient (ICC) as an assessment of the inter-session repeatability of the measurements for all sectors in the SCP and DCP.

### Vessel Density Measurement

**Figure 1:**
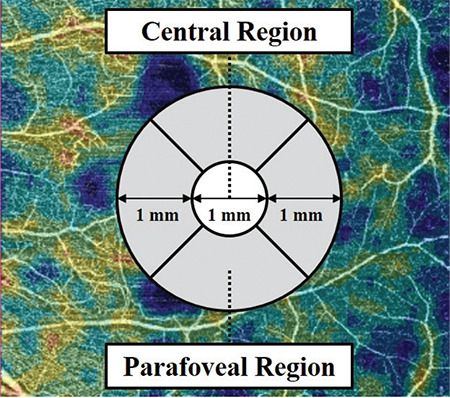
Schematic diagram of the 3 mm ETDRS grid centered over the fovea. The central 1-mm sector (shown in white) is the central region. The parafoveal region is the area (shown in gray) between the central 1-mm sector and the boundary of the 3-mm grid. The vessel density of the parafoveal region is the mean of the vessel density of the 4 sectors surrounding the central region. The average vessel density of the SCP and DCP were calculated as the mean of the vessel density of the area encompassed by the entire grid ETDRS: Early treatment of diabetic retinopathy study, SCP: Superficial capillary plexus , DCP: Deep capillary plexus

The vessel density values were obtained from a 3-mm circular Early Treatment DR Study (ETDRS) grid centered over the fovea. Vessel density was calculated as the proportion of the measured area occupied by blood vessels at the level of the SCP and DCP. The grid displayed the vessel density of each of the sectors. The central region was defined as the central 1-mm sector of the ETDRS grid. The parafoveal region was defined as the intervening region from the central 1-mm sector to the 3-mm boundary of the ETDRS grid. The vessel density of the central, parafoveal regions, and entire 3-mm region at the levels of SCP and DCP were obtained. Figure 1 is a schematic diagram that indicates the relative locations of the central and parafoveal regions.

### Optical Coherence Tomography of the Macula

To assess CST, the Spectralis OCT (Heidelberg Engineering, Heidelberg, Germany) was used. A 25-line horizontal raster scan (20°x20°, 6.0x6.0 mm) centered on the fovea was performed, with 9 frames averaged in each OCT B-scan. The CST was read off from the central 1-mm sector of the ETDRS grid centered over the fovea.

### Statistical Analysis

Statistical analysis was performed using the Statistical Package for the Social Sciences (IBM SPSS Statistics for Windows, Version 21.0; IBM Corp, Armonk, New York). Continuous variables were expressed as the mean ± standard deviation. Comparisons between groups were evaluated using the paired samples t test, chi-square test, or Fisher exact test where appropriate. A p value <0.05 was considered statistically significant.

## Results

A total of 22 eyes of 22 patients (10 males and 12 females) were studied. The average age was 53.6±8.0 years. At the point of diagnosis, all 22 eyes had center-involving DME with DR at different clinical stages (13 eyes had mild non-proliferative DR, 4 eyes had moderate non-proliferative DR, 5 eyes had severe non-proliferative DR). The mean follow-up time was 96.0±8.0 days. As treatment, 20 eyes received monthly intravitreal bevacizumab and 2 eyes received monthly intravitreal aflibercept.

Table 1 shows the CST and vessel density for the entire study population at baseline and after treatment. Overall, there were no significant differences in SCP or DCP vessel density in the central and parafoveal regions after treatment compared to baseline, while CST decreased from 416.5 µm to 331.2 µm (p=0.025).

The eyes were subsequently categorized according to anatomical response: 12 eyes were considered responders and 10 eyes were considered as non-responders. There were no significant differences in the age (54.2±7.6 vs 52.8±8.9 years, p=0.695), gender (7 vs 6 females, p=0.938), and follow-up time (97.6±7.8 vs 94.1±8.3 days, p=0.321) between the responders and non-responders. CST and vessel density of the SCP and DCP also did not differ significantly between the responders and non-responders at baseline (p>0.05).

After treatment, CST decreased by 173.7 µm in responders and increased by 20.8 µm in non-responders (p<0.0001). There were no corresponding significant differences in vessel density or changes therein between the responders and non-responders in the SCP and DCP after treatment. Table 2 shows the CST and vessel density of the responders and non-responders at baseline and after treatment.

**Figure 2:**
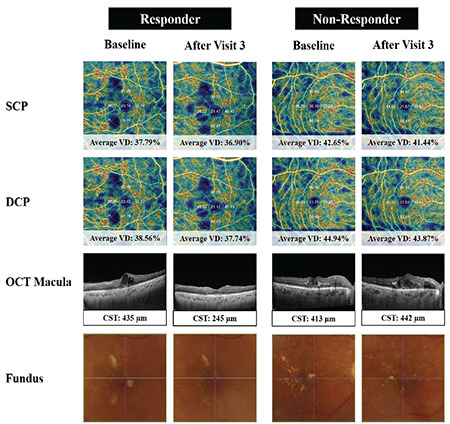
Serial multimodal images of a responder and non-responder. The vessel density (VD) of the superficial capillary plexus (SCP) and deep capillary plexus (DCP), optical coherence tomography (OCT) images of the macula, and fundus photographs at baseline and after the third visit are shown. Responder: Though the responder demonstrated anatomical improvement with a decrease in central subfield thickness, intraretinal fluid, subretinal fluid, and cystic spaces, there was no significant corresponding change in the vessel density in the SCP and DCP. Non-responder: The non-responder demonstrated anatomical worsening. Similarly, there was also no significant corresponding change in the vessel density in the SCP and DCP

Figure 2 shows serial multimodal images of a responder and non-responder. These images demonstrate the lack of corresponding change in vessel density in the SCP and DCP regardless of the anatomical response in the retina after VEGF inhibitor treatment.

The automatic segmentation lines, particularly over the areas affected by DME, had to be readjusted for all eyes in this study. Inter-session repeatability of the measurements was good for all sectors in the SCP and DCP (ICC =0.96 and 0.85, respectively).

## Discussion

In this pilot observational study which involved detailed manual segmentations of OCTA scans to evaluate macular vessel density in DME, the macular vessel density of the SCP and DCP were evaluated after 3 consecutive treatments of VEGF inhibitors. The vessel density and its changes were subsequently compared between anatomical responders and non-responders as defined by the CST change. We demonstrated that there were no significant changes in macular vessel density after VEGF inhibitor treatment and no relationship between macular vessel density and CST.

The previous studies describing longitudinal changes in vessel density after treatment reported conflicting results.^16,17,18,19,20,21,22^ Three studies demonstrated no significant differences in vessel density measured on OCTA after intravitreal injections despite improvement in edema and CST.^17,19,25^ These findings are similar to those of the current study. Several reasons have been postulated to explain this. Firstly, the retinal vessels which sustain ischemic damage in DME may not recover and perfuse after VEGF inhibition.^17^ Secondly, the displacement of the vessel plexus secondary to cystoid spaces in DME may only displace the retinal vessels without causing additional loss, hence the unchanged vessel density after resolution of the fluid and cystic spaces following treatment.^15^ The absence of significant change can also be attributed to the limitation and inaccuracy of automatic segmentation in OCTA as a result of anatomical distortion of the retinal layers in DME.^17,18,19^

Our findings were not confounded by segmentation inaccuracies because of our meticulous manual adjustment of the segmentation lines with the resultant good inter-session repeatability. The decrease in CST among responders supports previous findings that VEGF inhibitors reduce macular leakage by targeting VEGF and decreasing vessel hyperpermeability.^2^ However, the lack of corresponding change in the vessel density in the SCP and DCP regardless of the anatomical response of the retina after VEGF inhibitor treatment indicates that the effect of VEGF inhibitors in DME treatment may not be related to increasing vessel density. Any improvement of macular ischemia, therefore, may be an indirect effect of improved tissue perfusion and nutrition and not necessarily due to significant changes in the retinal vasculature.^25^

In contrast, other studies have reported a relationship between macular vessel density and response to DME treatment. A study reported that vessel density in the DCP, but not the SCP, was significantly increased after 12 months subsequent to the initial resolution of DME.^20^ There was also a study which reported that vessel density in the central region decreased by 8% after 3 aflibercept injections but remained unchanged in the parafoveal region.^24^ It was also reported that certain eyes may not respond to VEGF inhibitors and demonstrate a lower vessel density in the DCP but not the SCP.^16,20^ Another study reported that the vessel density of the SCP and DCP in the inner and outer parafovea increased significantly after 3 ranibizumab injections, but did not return to the normal levels.^22^

In comparison, we demonstrated that there were no significant changes in the macular vessel density of the SCP and DCP after the VEGF inhibitor treatment and there was no relationship between macular vessel density and CST. The inconsistency in findings among different studies can be attributed to differences in study populations, baseline characteristics, treatment, follow-up time, and imaging modalities used. See Table 3 for a comparison among studies. Of note, the criterion for response to VEGF inhibitor treatment used is also different. Two studies defined response by a reduction of more than 50 µm in CST after 3 consecutive anti-VEGF treatments.^16,20^ Therefore, responders which were defined as such might have been a subgroup with a very robust response to VEGF inhibitor treatment.^25^ In contrast, we defined response anatomically as a 10% decrease in CST from baseline.^33,34^

The mechanisms supporting an association between the improvement in the DCP and treatment response are also not clearly defined.^16,20^ A suggestion is that retinal fluid production originates from the SCP and is absorbed through Müller cells and the DCP in normal eyes.^35^ Hence, a recovery in the DCP could theoretically help resolve the edema in DME. Another possible explanation is that an improvement in the DCP will decrease the drive for VEGF production and aid the response to VEGF inhibitors.^16,20^

Separately, the observations in this study also agree with previous studies that demonstrated that VEGF inhibitors do not worsen retinal capillary nonperfusion.^36^ The link between ischemia and the administration of VEGF inhibitors has been investigated with other imaging modalities.^27^ Previous case series reported an increased risk of worsening of retinal nonperfusion in eyes with retinal vascular disease following the administration of VEGF inhibitors.^37^ These studies attributed the worsening of retinal nonperfusion to the blockage of VEGF, which is a survival factor for vascular endothelial cells.

A strength of this study is the meticulous manual segmentation of the automatic segmentation lines that were erroneous due to the disruption of anatomy in DME. The majority of the automatic segmentation lines, particularly over areas affected by the DME, had to be readjusted for all eyes. This process was performed twice, and the inter-session repeatability of the measurements was good. Another strength is the longitudinal design with the same number of treatments. In addition, the use of the in-built vessel density measurement tool ensured that this technique could be applied in clinical practice without complex image analysis.

### Study Limitations

There are several limitations in this study. It was retrospective with a small sample size, which may have made it difficult to detect small but significant changes in vessel density. The follow-up period was relatively short, and this may not have allowed for enough time to detect vessel density changes which may have manifested with long-term treatment. This study also included eyes with DR of different severities and treated with different VEGF inhibitors. The capillary response and vessel density changes with each VEGF inhibitor may differ. Averaging the vessel density in the central 3 mm of the ETDRS grid may have resulted in the loss of detection of focal areas of change in vessel density and FA was not performed to confirm the presence of ischemia. Although poor quality images were excluded and the segmentation lines were manually corrected, there is still a possibility of measurement error due to projection artifacts on OCTA that may also have confounded the results. This study was also dependent on manual segmentation of the layers on OCTA to overcome the issues of inaccurate segmentation and difficulty in obtaining vascular quantification as a result of distorted anatomy in diseased states. This was very labor-intensive. However, other methods currently involve custom image processing software that is usually unavailable in clinical settings.

## Conclusion

There were no significant changes in macular vessel density after the early stages of VEGF inhibitor treatment for DME, and there was no relationship with anatomical response. The effect of VEGF inhibitors in DME treatment therefore may not be directly related to increasing vessel density. This is a small pilot study with manual segmentation of each OCTA scan to overcome the issues of inaccurate segmentation and difficulty in obtaining vascular quantification as a result of distorted anatomy in diseased states. Further studies with larger population size and longer duration are needed to exposure the role of OCTA vessel density measurements as a potential biomarker of response to VEGF inhibitor treatment for DME.

## Figures and Tables

**Table 1:**
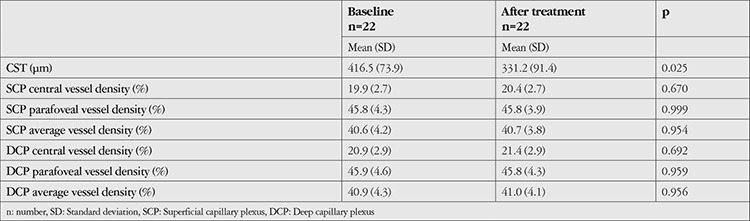
Central subfield thickness (CST) and vessel density in the study population at baseline and after treatment

**Table 2:**
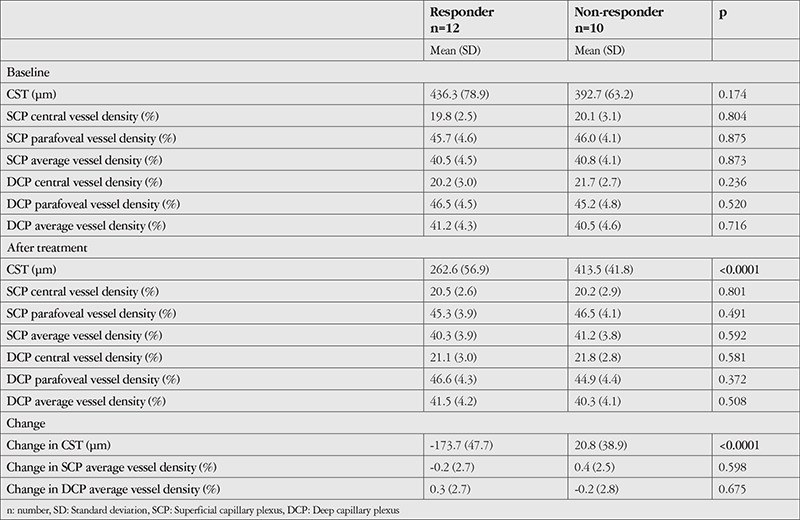
Central subfield thickness (CST) and vessel density at baseline and after treatment categorized by anatomical response

**Table 3:**
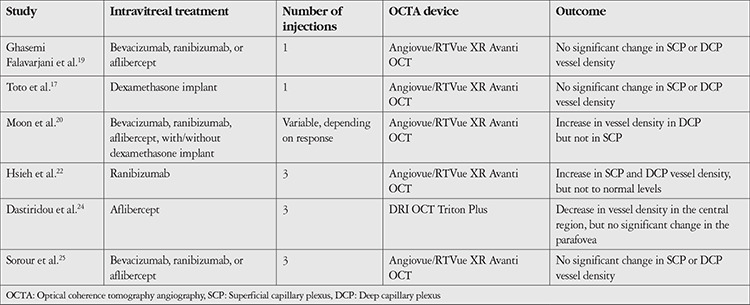
Comparison of studies
